# Ethane-1,2-diaminium 3,4,5,6-tetra­bromo-2-(methoxy­carbon­yl)benzoate methanol solvate

**DOI:** 10.1107/S1600536808038166

**Published:** 2008-11-22

**Authors:** Zu-Pei Liang

**Affiliations:** a Department of Chemistry and Chemical Engineering, Weifang University, Weifang 261061, People’s Republic of China

## Abstract

In the title compound, C_2_H_10_N_2_^2+^·2C_9_H_3_Br_4_O_4_^−^·CH_4_O, the N atoms of the ethane-1,2-diamine mol­ecule are protonated. The crystal structure is stabilized by N—H⋯O hydrogen bonds between the ethane-1,2-diaminium cations and 3,4,5,6-tetra­bromo-2-(methoxy­carbon­yl)bromo­benzoate anions, and by O—H⋯O and N—H⋯O hydrogen bonds between the methanol solvate and both the cation and the anion. In addition, the crystal structure exhibits a C—Br⋯O halogen bond [3.20 (3) Å] and a Br⋯Br inter­action [3.560 (2) Å].

## Related literature

For related structures, see: Liang *et al.* (2006 ▶, 2007 ▶); For a review of halogen bonding, see: Politzer *et al.* (2007 ▶).
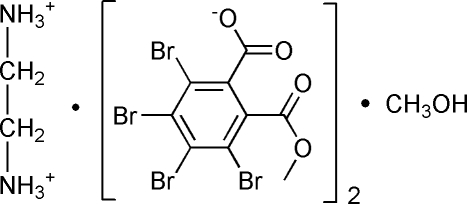


## Experimental

### 

#### Crystal data

C_2_H_10_N_2_^2+^·2C_9_H_3_Br_4_O_4_^−^·CH_4_O
*M* *_r_* = 1083.67
Monoclinic, 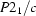

*a* = 6.456 (2) Å
*b* = 19.036 (7) Å
*c* = 26.017 (9) Å
β = 96.002 (6)°
*V* = 3179.7 (19) Å^3^
*Z* = 4
Mo *K*α radiation
μ = 10.14 mm^−1^
*T* = 298 (2) K
0.41 × 0.25 × 0.15 mm

#### Data collection

Bruker SMART CCD area-detector diffractometer
Absorption correction: multi-scan (*SADABS*; Bruker, 1997 ▶) *T*_min_ = 0.062, *T*_max_ = 0.221
15827 measured reflections
5585 independent reflections
3448 reflections with *I* > 2σ(*I*)
*R*_int_ = 0.083

#### Refinement

*R*[*F* ^2^ > 2σ(*F* ^2^)] = 0.055
*wR*(*F* ^2^) = 0.145
*S* = 0.98
5585 reflections
367 parameters
6 restraints
H-atom parameters constrained
Δρ_max_ = 1.18 e Å^−3^
Δρ_min_ = −0.72 e Å^−3^

### 

Data collection: *SMART* (Bruker, 1997 ▶); cell refinement: *SAINT* (Bruker, 1997 ▶); data reduction: *SAINT*; program(s) used to solve structure: *SHELXS97* (Sheldrick, 2008 ▶); program(s) used to refine structure: *SHELXL97* (Sheldrick, 2008 ▶); molecular graphics: *SHELXTL* (Sheldrick, 2008 ▶) and *DIAMOND* (Brandenburg, 1998 ▶); software used to prepare material for publication: *SHELXTL*.

## Supplementary Material

Crystal structure: contains datablocks global, I. DOI: 10.1107/S1600536808038166/lx2065sup1.cif

Structure factors: contains datablocks I. DOI: 10.1107/S1600536808038166/lx2065Isup2.hkl

Additional supplementary materials:  crystallographic information; 3D view; checkCIF report

## Figures and Tables

**Table 1:** Hydrogen-bond geometry (Å, °)

*D*—H⋯*A*	*D*—H	H⋯*A*	*D*⋯*A*	*D*—H⋯*A*
N1—H1*A*⋯O2^i^	0.89	1.87	2.748 (8)	167
N1—H1*B*⋯O5^ii^	0.89	2.04	2.857 (8)	153
N1—H1*C*⋯O6	0.89	1.86	2.734 (8)	166
N2—H2*A*⋯O9^iii^	0.89	1.97	2.801 (9)	154
N2—H2*B*⋯O2^iii^	0.89	1.93	2.795 (9)	163
N2—H2*B*⋯O3^iii^	0.89	2.57	3.000 (9)	110
N2—H2*C*⋯O5^iv^	0.89	1.90	2.747 (9)	159
O9—H9⋯O1^v^	0.82	1.89	2.695 (8)	168

Symmetry codes: (i) 
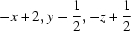
; (ii) 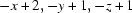; (iii) 
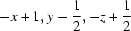
; (iv) 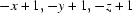; (v) 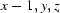.

**Fig. 1.**
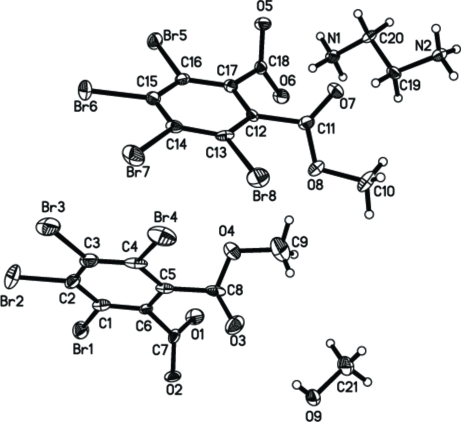
The molecular structure of (I), drawn with 30% probability ellipsoids.

**Fig. 2.**
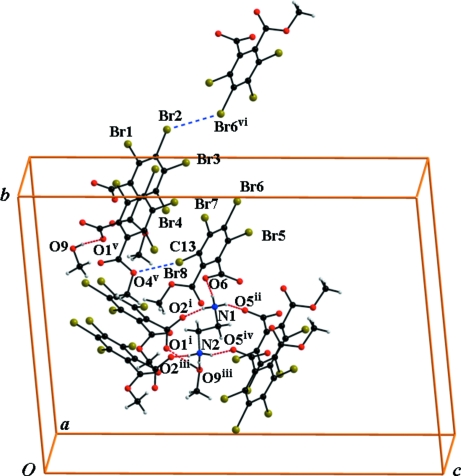
The crystal packing of (I), viewed along a axis. Hydrogen bonds are indicated by dashed lines. [Symmetry code: (i) -x + 2, y - 1/2, -z + 1/2; (ii) -x + 2, -y + 1, -z + 1; (iii) -x + 1, y - 1/2, -z + 1/2; (iv) -x + 1, -y + 1, -z + 1; (v) x - 1, y, z; (vi) -x + 1, -y + 2, -z + 1.]

## supplementary crystallographic information

### Comment

1,2-Bis(tetrabromophthalimido)ethane is an important flame retardant. 2-(Methoxycarbonyl)-3,4,5,6-tetrabromobenzoic acid is the intermediate of the flame retardant. In this paper, the structure of the title compound is reported.

The asymmetric unit of the title compound contains one ethane-1,2-diaminium, two 3,4,5,6-tetrabromo-2-(methoxycarbonyl)bromobenzoate and one methanol molecule (Fig. 1). The bond lengths and angles agree with those in those similar compounds 4-phthalimidobenzoic acid *N*,*N*-dimethylformamide solvate (Liang *et al.*, 2006) and 4-(5-Bromo-1,3-dioxoisoindolin-2-yl)benzoic acid *N*,*N*-dimethylformamide solvate (Liang *et al.*, 2007). The dihedral angle between two benzene rings is 74.6 (2)°.

The crystal structure is stabilized by various hydrogen bonds (Fig. 2 and Table 1; symmetry code as in Fig. 2); N—H···O hydrogen bonds between the ethane-1,2-diaminium and the 2-(methoxycarbonyl)-3,4,5,6-tetrabromobenzoate anions, O—H···O and N—H···O hydrogen bonds between the methanol and the 3,4,5,6-tetrabromo-2-(methoxycarbonyl)bromobenzoate anions and the ethane-1,2-diaminium. The further stability comes from a weak C—Br···O halogen bond (Fig. 2) (Politzer *et al.*, 2007); between the bromine atom and the oxygen of a neighbouring methoxy group, *i.e.* C13—Br8···O4^v^ distance of 3.20 (3) Å and a C13—Br8···O4^v^ angle of 156.9 (3)°, and a Br2···Br6^vi^ interaction at 3.560 (2) Å (Fig. 2).

### Experimental

A mixture of 4,5,6,7-tetrabromoisobenzofuran-1,3-dione (46.4 g, 0.1 mol) and methanol (150 ml) was refluxed for 0.5 h. And then ethane-1,2-diamine (3 g, 0.05 mol) was added to the above solution, being mixed round for 4 h at room temperature. After filtration and the filtrate was kept at room temperature for 3 d. Natural evaporation gave colourless single crystals of the title compound, suitable for X-ray analysis.

### Refinement

H atoms were initially located from difference maps and then refined in a riding model with C—H = 0.93–0.96 Å, N—H = 0.89 Å and *U*_iso_(H) = 1.2*U*_eq_(C, N) or 1.5*U*_eq_(O, methyl C).

### Crystal data

**Table:**

C_2_H_10_N_2_^2+^·2C_9_H_3_Br_4_O_4_^–^·CH_4_O	*F*_000_ = 2048
*M**_r_* = 1083.67	*D*_x_ = 2.264 Mg m^−^^3^
Monoclinic, *P*2_1_/*c*	Mo *K*α radiation λ = 0.71073 Å
Hall symbol: -P 2ybc	Cell parameters from 2716 reflections
*a* = 6.456 (2) Å	θ = 2.3–22.1º
*b* = 19.036 (7) Å	µ = 10.14 mm^−^^1^
*c* = 26.017 (9) Å	*T* = 298 (2) K
β = 96.002 (6)º	Block, colourless
*V* = 3179.7 (19) Å^3^	0.41 × 0.25 × 0.15 mm
*Z* = 4	

### Data collection

**Table:**

Bruker SMART CCD area-detector diffractometer	5585 independent reflections
Radiation source: fine-focus sealed tube	3448 reflections with *I* > 2σ(*I*)
Monochromator: graphite	*R*_int_ = 0.083
Detector resolution: 10.0 pixels mm^-1^	θ_max_ = 25.0º
*T* = 298(2) K	θ_min_ = 1.6º
φ and ω scans	*h* = −7→6
Absorption correction: multi-scan(SADABS; Bruker, 1997)	*k* = −22→22
*T*_min_ = 0.062, *T*_max_ = 0.221	*l* = −30→25
15827 measured reflections	

### Refinement

**Table:**

Refinement on *F*^2^	Secondary atom site location: difference Fourier map
Least-squares matrix: full	Hydrogen site location: inferred from neighbouring sites
*R*[*F*^2^ > 2σ(*F*^2^)] = 0.055	H-atom parameters constrained
*wR*(*F*^2^) = 0.145	*w* = 1/[σ^2^(*F*_o_^2^) + (0.0474*P*)^2^] where *P* = (*F*_o_^2^ + 2*F*_c_^2^)/3
*S* = 0.98	(Δ/σ)_max_ < 0.001
5585 reflections	Δρ_max_ = 1.18 e Å^−^^3^
367 parameters	Δρ_min_ = −0.72 e Å^−^^3^
6 restraints	Extinction correction: none
Primary atom site location: structure-invariant direct methods	

### Special details

**Table:**

Geometry. All e.s.d.'s (except the e.s.d. in the dihedral angle between two l.s. planes) are estimated using the full covariance matrix. The cell e.s.d.'s are taken into account individually in the estimation of e.s.d.'s in distances, angles and torsion angles; correlations between e.s.d.'s in cell parameters are only used when they are defined by crystal symmetry. An approximate (isotropic) treatment of cell e.s.d.'s is used for estimating e.s.d.'s involving l.s. planes.
Refinement. Refinement of *F*^2^ against ALL reflections. The weighted *R*-factor *wR* and goodness of fit *S* are based on *F*^2^, conventional *R*-factors *R* are based on *F*, with *F* set to zero for negative *F*^2^. The threshold expression of *F*^2^ > σ(*F*^2^) is used only for calculating *R*-factors(gt) *etc*. and is not relevant to the choice of reflections for refinement. *R*-factors based on *F*^2^ are statistically about twice as large as those based on *F*, and *R*- factors based on ALL data will be even larger.

### Fractional atomic coordinates and isotropic or equivalent isotropic displacement parameters (Å^2^)

**Table:**

	*x*	*y*	*z*	*U*_iso_*/*U*_eq_	
C13	0.2706 (12)	0.7587 (4)	0.4305 (3)	0.028 (2)	
Br5	0.90144 (14)	0.74383 (5)	0.53528 (4)	0.0387 (3)	
Br8	0.01592 (15)	0.76205 (5)	0.38806 (4)	0.0515 (3)	
Br7	0.18589 (16)	0.89882 (5)	0.45626 (4)	0.0508 (3)	
Br1	1.30682 (16)	1.00363 (5)	0.23756 (4)	0.0488 (3)	
Br6	0.64413 (16)	0.89277 (5)	0.52383 (4)	0.0473 (3)	
Br3	0.7771 (2)	1.01282 (6)	0.39936 (4)	0.0712 (4)	
Br4	0.52006 (19)	0.87733 (7)	0.34731 (5)	0.0694 (4)	
Br2	1.1722 (2)	1.07590 (7)	0.34433 (5)	0.0789 (4)	
O5	0.7309 (8)	0.5905 (3)	0.5074 (2)	0.0316 (14)	
O6	0.7401 (9)	0.6008 (3)	0.4220 (2)	0.0355 (15)	
O2	0.9487 (9)	0.9138 (3)	0.1572 (2)	0.0366 (15)	
O9	0.2812 (10)	0.7719 (3)	0.1141 (2)	0.0387 (15)	
H9	0.2528	0.7917	0.1405	0.058*	
C15	0.5347 (13)	0.8125 (4)	0.4890 (3)	0.029 (2)	
N1	0.9242 (10)	0.4714 (3)	0.4311 (2)	0.0295 (17)	
H1A	0.9667	0.4597	0.4008	0.044*	
H1B	1.0283	0.4655	0.4559	0.044*	
H1C	0.8841	0.5161	0.4303	0.044*	
C14	0.3451 (13)	0.8158 (4)	0.4590 (3)	0.027 (2)	
C4	0.7531 (14)	0.9129 (5)	0.3182 (3)	0.040 (2)	
C5	0.8078 (13)	0.8814 (4)	0.2718 (3)	0.031 (2)	
C11	0.3057 (13)	0.6319 (5)	0.4029 (3)	0.031 (2)	
O1	1.1336 (11)	0.8281 (3)	0.1981 (2)	0.0498 (18)	
C16	0.6458 (12)	0.7499 (4)	0.4910 (3)	0.0247 (18)	
C19	0.5754 (12)	0.4323 (4)	0.3977 (3)	0.029 (2)	
H19A	0.5067	0.4775	0.3995	0.035*	
H19B	0.6339	0.4295	0.3649	0.035*	
C17	0.5723 (12)	0.6915 (4)	0.4644 (3)	0.0234 (19)	
N2	0.4214 (10)	0.3748 (3)	0.4012 (2)	0.0307 (17)	
H2A	0.4809	0.3337	0.3956	0.046*	
H2B	0.3132	0.3815	0.3776	0.046*	
H2C	0.3779	0.3748	0.4326	0.046*	
O4	0.7137 (11)	0.7667 (3)	0.2822 (3)	0.059 (2)	
C18	0.6933 (12)	0.6221 (4)	0.4644 (3)	0.0236 (19)	
O3	0.6344 (11)	0.8134 (4)	0.2056 (3)	0.061 (2)	
C1	1.0757 (13)	0.9683 (5)	0.2702 (3)	0.034 (2)	
C6	0.9726 (13)	0.9107 (4)	0.2478 (3)	0.0260 (19)	
C12	0.3863 (12)	0.6964 (4)	0.4321 (3)	0.0212 (18)	
C7	1.0268 (14)	0.8808 (5)	0.1962 (3)	0.030 (2)	
O7	0.2565 (9)	0.5792 (3)	0.4236 (2)	0.0381 (15)	
O8	0.2970 (10)	0.6420 (3)	0.3525 (2)	0.0475 (17)	
C8	0.7027 (14)	0.8161 (5)	0.2485 (4)	0.039 (2)	
C2	1.0236 (15)	0.9986 (5)	0.3156 (3)	0.039 (2)	
C3	0.8613 (17)	0.9711 (5)	0.3390 (4)	0.046 (3)	
C20	0.7472 (12)	0.4262 (4)	0.4415 (3)	0.029 (2)	
H20A	0.6956	0.4404	0.4736	0.035*	
H20B	0.7929	0.3777	0.4450	0.035*	
C21	0.3236 (18)	0.7007 (5)	0.1248 (4)	0.059 (3)	
H21A	0.4547	0.6966	0.1457	0.089*	
H21B	0.3300	0.6757	0.0930	0.089*	
H21C	0.2153	0.6813	0.1431	0.089*	
C10	0.192 (2)	0.5888 (6)	0.3205 (4)	0.077 (4)	
H10A	0.2580	0.5442	0.3279	0.116*	
H10B	0.1982	0.6004	0.2848	0.116*	
H10C	0.0488	0.5861	0.3274	0.116*	
C9	0.605 (3)	0.7038 (7)	0.2652 (5)	0.112 (6)	
H9A	0.4793	0.7160	0.2440	0.169*	
H9B	0.5708	0.6776	0.2946	0.169*	
H9C	0.6919	0.6759	0.2455	0.169*	

### Atomic displacement parameters (Å^2^)

**Table:**

	*U*^11^	*U*^22^	*U*^33^	*U*^12^	*U*^13^	*U*^23^
C13	0.026 (5)	0.040 (5)	0.017 (4)	0.004 (4)	−0.001 (3)	0.010 (4)
Br5	0.0371 (5)	0.0371 (6)	0.0384 (6)	−0.0028 (4)	−0.0124 (4)	−0.0011 (4)
Br8	0.0390 (6)	0.0536 (7)	0.0570 (7)	0.0077 (5)	−0.0170 (5)	0.0117 (5)
Br7	0.0594 (7)	0.0334 (6)	0.0609 (7)	0.0211 (5)	0.0124 (5)	0.0069 (5)
Br1	0.0489 (6)	0.0493 (6)	0.0484 (7)	−0.0134 (5)	0.0065 (5)	−0.0022 (5)
Br6	0.0684 (7)	0.0281 (5)	0.0453 (6)	−0.0054 (5)	0.0055 (5)	−0.0119 (5)
Br3	0.1170 (11)	0.0641 (8)	0.0366 (6)	0.0414 (7)	0.0267 (6)	0.0001 (6)
Br4	0.0671 (8)	0.0844 (9)	0.0636 (8)	0.0141 (6)	0.0398 (6)	0.0286 (7)
Br2	0.1045 (11)	0.0677 (8)	0.0625 (8)	−0.0110 (7)	−0.0005 (7)	−0.0366 (7)
O5	0.038 (4)	0.032 (3)	0.025 (3)	0.006 (3)	0.003 (3)	0.004 (3)
O6	0.050 (4)	0.026 (3)	0.032 (4)	0.010 (3)	0.011 (3)	−0.001 (3)
O2	0.046 (4)	0.047 (4)	0.017 (3)	−0.008 (3)	0.003 (3)	0.009 (3)
O9	0.053 (4)	0.026 (3)	0.036 (4)	−0.003 (3)	0.005 (3)	−0.002 (3)
C15	0.044 (6)	0.022 (5)	0.023 (5)	0.004 (4)	0.008 (4)	0.000 (4)
N1	0.037 (4)	0.029 (4)	0.023 (4)	0.005 (3)	0.007 (3)	−0.004 (3)
C14	0.034 (5)	0.023 (5)	0.023 (5)	0.011 (4)	0.008 (4)	0.005 (4)
C4	0.037 (6)	0.059 (7)	0.027 (5)	0.022 (5)	0.012 (4)	0.023 (5)
C5	0.033 (5)	0.041 (6)	0.021 (5)	0.008 (4)	0.006 (4)	0.013 (4)
C11	0.034 (5)	0.037 (6)	0.023 (5)	−0.002 (4)	−0.001 (4)	0.001 (4)
O1	0.065 (5)	0.046 (4)	0.039 (4)	0.025 (4)	0.006 (3)	−0.006 (3)
C16	0.028 (4)	0.028 (5)	0.017 (4)	0.000 (4)	0.001 (3)	0.003 (4)
C19	0.037 (5)	0.021 (5)	0.030 (5)	0.001 (4)	0.005 (4)	0.006 (4)
C17	0.027 (5)	0.021 (5)	0.023 (5)	0.003 (3)	0.006 (4)	0.001 (4)
N2	0.033 (4)	0.035 (4)	0.022 (4)	0.000 (3)	−0.003 (3)	0.001 (3)
O4	0.078 (5)	0.043 (4)	0.055 (5)	−0.021 (4)	0.000 (4)	0.012 (4)
C18	0.022 (4)	0.022 (5)	0.026 (5)	−0.002 (3)	−0.002 (4)	0.000 (4)
O3	0.061 (5)	0.083 (6)	0.038 (4)	−0.036 (4)	0.002 (4)	−0.001 (4)
C1	0.034 (5)	0.046 (6)	0.023 (5)	0.009 (4)	0.004 (4)	0.008 (4)
C6	0.037 (5)	0.024 (5)	0.016 (4)	0.009 (4)	−0.002 (4)	0.001 (4)
C12	0.028 (5)	0.014 (4)	0.022 (5)	0.000 (3)	0.003 (4)	0.003 (3)
C7	0.037 (5)	0.034 (5)	0.021 (5)	−0.013 (4)	0.008 (4)	−0.008 (4)
O7	0.048 (4)	0.026 (3)	0.038 (4)	−0.004 (3)	−0.001 (3)	0.009 (3)
O8	0.069 (5)	0.044 (4)	0.026 (4)	−0.011 (3)	−0.011 (3)	−0.003 (3)
C8	0.039 (5)	0.053 (5)	0.025 (4)	−0.005 (4)	0.013 (4)	0.023 (4)
C2	0.051 (6)	0.040 (6)	0.025 (5)	0.009 (5)	−0.003 (4)	−0.007 (5)
C3	0.064 (7)	0.043 (6)	0.029 (6)	0.018 (5)	0.000 (5)	0.004 (5)
C20	0.034 (5)	0.037 (5)	0.015 (4)	0.002 (4)	0.001 (4)	−0.006 (4)
C21	0.096 (9)	0.028 (6)	0.052 (7)	−0.001 (6)	0.001 (6)	−0.007 (5)
C10	0.114 (11)	0.071 (9)	0.040 (7)	−0.029 (7)	−0.024 (7)	−0.014 (6)
C9	0.174 (17)	0.064 (9)	0.097 (11)	−0.052 (10)	0.006 (10)	0.011 (8)

### Geometric parameters (Å, °)

**Table:**

C13—C14	1.375 (11)	C16—C17	1.369 (10)
C13—C12	1.400 (10)	C19—N2	1.489 (10)
C13—Br8	1.883 (8)	C19—C20	1.509 (10)
Br5—C16	1.915 (8)	C19—H19A	0.9700
Br7—C14	1.882 (7)	C19—H19B	0.9700
Br1—C1	1.914 (9)	C17—C12	1.394 (11)
Br6—C15	1.877 (8)	C17—C18	1.533 (11)
Br3—C3	1.891 (10)	N2—H2A	0.8900
Br4—C4	1.880 (9)	N2—H2B	0.8900
Br2—C2	1.869 (9)	N2—H2C	0.8900
O5—C18	1.272 (9)	O4—C8	1.284 (10)
O6—C18	1.240 (9)	O4—C9	1.434 (13)
O2—C7	1.253 (10)	O3—C8	1.158 (10)
O9—C21	1.404 (10)	C1—C6	1.381 (11)
O9—H9	0.8200	C1—C2	1.387 (12)
C15—C14	1.382 (11)	C6—C7	1.532 (11)
C15—C16	1.389 (10)	O8—C10	1.436 (10)
N1—C20	1.478 (10)	C2—C3	1.369 (13)
N1—H1A	0.8900	C20—H20A	0.9700
N1—H1B	0.8900	C20—H20B	0.9700
N1—H1C	0.8900	C21—H21A	0.9600
C4—C3	1.388 (13)	C21—H21B	0.9600
C4—C5	1.424 (12)	C21—H21C	0.9600
C5—C6	1.405 (11)	C10—H10A	0.9600
C5—C8	1.512 (13)	C10—H10B	0.9600
C11—O7	1.197 (9)	C10—H10C	0.9600
C11—O8	1.321 (10)	C9—H9A	0.9600
C11—C12	1.506 (11)	C9—H9B	0.9600
O1—C7	1.215 (10)	C9—H9C	0.9600
			
?···?	?		
			
C14—C13—C12	119.9 (7)	C6—C1—C2	122.7 (8)
C14—C13—Br8	121.6 (6)	C6—C1—Br1	117.1 (6)
C12—C13—Br8	118.5 (6)	C2—C1—Br1	120.1 (7)
C21—O9—H9	109.5	C1—C6—C5	118.8 (8)
C14—C15—C16	118.9 (7)	C1—C6—C7	121.4 (8)
C14—C15—Br6	120.1 (6)	C5—C6—C7	119.7 (7)
C16—C15—Br6	120.9 (6)	C17—C12—C13	119.9 (7)
C20—N1—H1A	109.5	C17—C12—C11	118.6 (7)
C20—N1—H1B	109.5	C13—C12—C11	121.3 (7)
H1A—N1—H1B	109.5	O1—C7—O2	128.7 (8)
C20—N1—H1C	109.5	O1—C7—C6	116.8 (8)
H1A—N1—H1C	109.5	O2—C7—C6	114.4 (8)
H1B—N1—H1C	109.5	C11—O8—C10	116.3 (8)
C13—C14—C15	120.5 (7)	O3—C8—O4	127.9 (10)
C13—C14—Br7	119.0 (6)	O3—C8—C5	122.3 (8)
C15—C14—Br7	120.5 (6)	O4—C8—C5	109.6 (8)
C3—C4—C5	120.4 (9)	C3—C2—C1	119.0 (9)
C3—C4—Br4	121.5 (7)	C3—C2—Br2	120.5 (7)
C5—C4—Br4	118.0 (8)	C1—C2—Br2	120.5 (8)
C6—C5—C4	118.5 (8)	C2—C3—C4	120.6 (9)
C6—C5—C8	118.4 (7)	C2—C3—Br3	120.6 (8)
C4—C5—C8	123.1 (8)	C4—C3—Br3	118.8 (8)
O7—C11—O8	125.6 (8)	N1—C20—C19	109.7 (6)
O7—C11—C12	123.4 (8)	N1—C20—H20A	109.7
O8—C11—C12	111.0 (7)	C19—C20—H20A	109.7
C17—C16—C15	121.9 (7)	N1—C20—H20B	109.7
C17—C16—Br5	119.1 (6)	C19—C20—H20B	109.7
C15—C16—Br5	118.9 (6)	H20A—C20—H20B	108.2
N2—C19—C20	109.9 (6)	O9—C21—H21A	109.5
N2—C19—H19A	109.7	O9—C21—H21B	109.5
C20—C19—H19A	109.7	H21A—C21—H21B	109.5
N2—C19—H19B	109.7	O9—C21—H21C	109.5
C20—C19—H19B	109.7	H21A—C21—H21C	109.5
H19A—C19—H19B	108.2	H21B—C21—H21C	109.5
C16—C17—C12	118.8 (7)	O8—C10—H10A	109.5
C16—C17—C18	123.3 (7)	O8—C10—H10B	109.5
C12—C17—C18	117.7 (7)	H10A—C10—H10B	109.5
C19—N2—H2A	109.5	O8—C10—H10C	109.5
C19—N2—H2B	109.5	H10A—C10—H10C	109.5
H2A—N2—H2B	109.5	H10B—C10—H10C	109.5
C19—N2—H2C	109.5	O4—C9—H9A	109.5
H2A—N2—H2C	109.5	O4—C9—H9B	109.5
H2B—N2—H2C	109.5	H9A—C9—H9B	109.5
C8—O4—C9	114.2 (9)	O4—C9—H9C	109.5
O6—C18—O5	125.7 (7)	H9A—C9—H9C	109.5
O6—C18—C17	117.0 (7)	H9B—C9—H9C	109.5
O5—C18—C17	117.2 (7)		
			
C12—C13—C14—C15	0.6 (12)	C18—C17—C12—C11	5.4 (11)
Br8—C13—C14—C15	−178.6 (6)	C14—C13—C12—C17	2.8 (12)
C12—C13—C14—Br7	−179.8 (6)	Br8—C13—C12—C17	−178.0 (6)
Br8—C13—C14—Br7	1.0 (9)	C14—C13—C12—C11	177.7 (7)
C16—C15—C14—C13	−1.9 (12)	Br8—C13—C12—C11	−3.1 (10)
Br6—C15—C14—C13	175.4 (6)	O7—C11—C12—C17	62.5 (11)
C16—C15—C14—Br7	178.6 (6)	O8—C11—C12—C17	−117.7 (8)
Br6—C15—C14—Br7	−4.1 (10)	O7—C11—C12—C13	−112.4 (10)
C3—C4—C5—C6	−0.2 (12)	O8—C11—C12—C13	67.4 (10)
Br4—C4—C5—C6	175.6 (6)	C1—C6—C7—O1	101.9 (10)
C3—C4—C5—C8	177.3 (8)	C5—C6—C7—O1	−81.3 (10)
Br4—C4—C5—C8	−6.9 (11)	C1—C6—C7—O2	−79.9 (10)
C14—C15—C16—C17	−0.3 (13)	C5—C6—C7—O2	96.9 (9)
Br6—C15—C16—C17	−177.6 (6)	O7—C11—O8—C10	9.4 (13)
C14—C15—C16—Br5	−176.8 (6)	C12—C11—O8—C10	−170.4 (8)
Br6—C15—C16—Br5	5.9 (9)	C9—O4—C8—O3	−8.1 (16)
C15—C16—C17—C12	3.7 (12)	C9—O4—C8—C5	175.6 (10)
Br5—C16—C17—C12	−179.8 (6)	C6—C5—C8—O3	−54.1 (13)
C15—C16—C17—C18	178.1 (8)	C4—C5—C8—O3	128.5 (10)
Br5—C16—C17—C18	−5.4 (11)	C6—C5—C8—O4	122.5 (8)
C16—C17—C18—O6	−119.1 (9)	C4—C5—C8—O4	−55.0 (11)
C12—C17—C18—O6	55.3 (10)	C6—C1—C2—C3	−1.0 (13)
C16—C17—C18—O5	63.1 (11)	Br1—C1—C2—C3	−178.1 (7)
C12—C17—C18—O5	−122.5 (8)	C6—C1—C2—Br2	179.1 (6)
C2—C1—C6—C5	0.1 (12)	Br1—C1—C2—Br2	2.0 (10)
Br1—C1—C6—C5	177.3 (6)	C1—C2—C3—C4	1.3 (14)
C2—C1—C6—C7	176.9 (8)	Br2—C2—C3—C4	−178.8 (6)
Br1—C1—C6—C7	−5.9 (10)	C1—C2—C3—Br3	−177.4 (6)
C4—C5—C6—C1	0.5 (12)	Br2—C2—C3—Br3	2.4 (11)
C8—C5—C6—C1	−177.1 (7)	C5—C4—C3—C2	−0.7 (13)
C4—C5—C6—C7	−176.4 (7)	Br4—C4—C3—C2	−176.4 (7)
C8—C5—C6—C7	6.0 (12)	C5—C4—C3—Br3	178.0 (6)
C16—C17—C12—C13	−4.9 (12)	Br4—C4—C3—Br3	2.4 (10)
C18—C17—C12—C13	−179.6 (7)	N2—C19—C20—N1	166.0 (6)
C16—C17—C12—C11	−179.9 (7)		

### Hydrogen-bond geometry (Å, °)

**Table:**

*D*—H···*A*	*D*—H	H···*A*	*D*···*A*	*D*—H···*A*
N1—H1A···O2^i^	0.89	1.87	2.748 (8)	167
N1—H1B···O5^ii^	0.89	2.04	2.857 (8)	153
N1—H1C···O6	0.89	1.86	2.734 (8)	166
N2—H2A···O9^iii^	0.89	1.97	2.801 (9)	154
N2—H2B···O2^iii^	0.89	1.93	2.795 (9)	163
N2—H2B···O3^iii^	0.89	2.57	3.000 (9)	110
N2—H2C···O5^iv^	0.89	1.90	2.747 (9)	159
O9—H9···O1^v^	0.82	1.89	2.695 (8)	168

Symmetry codes: (i) −*x*+2, *y*−1/2, −*z*+1/2; (ii) −*x*+2, −*y*+1, −*z*+1; (iii) −*x*+1, *y*−1/2, −*z*+1/2; (iv) −*x*+1, −*y*+1, −*z*+1; (v) *x*−1, *y*, *z*.
